# Practical guide on home health in heart failure patients

**DOI:** 10.5334/ijic.992

**Published:** 2013-11-04

**Authors:** Tiny Jaarsma, Torben Larsen, Anna Strömberg

**Affiliations:** Department of Social and Welfare Studies, Linköping University, Linköping, Sweden; CAST, University of Southern Denmark, Odense, Denmark; Division of Nursing, Department of Medical and Health Sciences, Linköping University, Linköping, Sweden

**Keywords:** integrated care, home care, heart failure, multidisciplinary

## Abstract

**Introduction:**

Chronic heart failure is a common condition affecting up to 15 million people in the extended Europe. Heart failure is burdensome and costly for patients in terms of decreased quality of life and poor prognosis, and it is also costly for society. Better integrated care is warranted in this population and specialised heart failure care can save costs and improve the quality of care. However, only a few European countries have implemented specialised home care and offered this to a larger number of patients with heart failure.

**Method:**

We developed a guide on Home Health in Heart Failure patients from a literature review, a survey of heart failure management programs, the opinion of researchers and practitioners, data from clinical trials and a reflection of an international expert meeting.

**Results:**

In integrated home care for heart failure patients, it is advised to consider the following components: integrated multidisciplinary care, patient and partner participation, care plans with clear goals of care, patient education, self-care management, appropriate access to care and optimised treatment.

**Discussion:**

We summarised the state of the art of home-based care for heart failure patients in Europe, described the typical content of such care to provide a guide for health care providers.

## Background and purpose of this guide

The number of patients suffering from heart failure is increasing worldwide due to reduced mortality after acute myocardial infarction, improved heart failure treatment and an increased average length of life in the general population. In the adult population of developed countries approximately 1–2% has heart failure, rising to 10% among persons of 70 years of age or older [1]. Heart failure is one of the leading causes of hospitalisation among people above 65 years of age to a cost of almost 2% of the total health care budget in many European countries. The length of stay in hospitals due to deterioration of heart failure decreased, but many patients with heart failure are readmitted shortly after a previous care episode of heart failure [2]. Many patients do not receive optimal treatment and care through a heart failure management programme [3] as strongly recommended in both the European and American guidelines for heart failure care [1,2,4–6]. Although it is generally accepted that multidisciplinary management and follow-up of heart failure patients are effective to improve patient adherence, reduce hospital readmissions and improve survival [1,4,7], there is an ongoing discussion on the optimal model of heart failure management. Most heart failure management programmes aim at optimisation of both pharmacological and non-pharmacological management and include assessment and intervention of risks and co-morbidity, optimised medical management, device therapy (pacemaker, cardiac resyncronisation therapy and implantable cardioverter defibrillator) education and self-care management, follow-up, access to health care and psychosocial [1,4,7,8]. Currently, the most optimal model for heart failure management is not known. Recent large-scaled studies show that not all models are equally successful to improve the outcomes, and these results indicate that a sophisticated approach to heart failure management is needed [9,10]. In a meta-analysis of McAlister and colleagues [7], the effects of multidisciplinary heart failure clinics were separated from the programmes that provided specialised follow-up in a non-clinic setting. Programmes in a home-based setting were found to be as effective in reducing mortality and rehospitalisation as the clinic setting. In a recent post hoc analysis of the ‘WHICH?-study’, [11,12] home-based care was associated with reduction in hospital stay, including a 35% drop in all-cause-hospitalisation days and a 37% decrease in cardiovascular-hospitalisation days, compared with clinic-based management.

Since the mean age of patients with heart failure is high (above 75 years) and patients often suffer from fatigue, disabilities and poor social support, it has been suggested in many European countries that integrated home care is an alternative form of care. Heart failure management, such as home care programmes, need to be adapted to the health care system and adapted to national priorities, with regard to financial resources, available personnel, administrative policies, infrastructure and tailored to patient needs. However, only in a few European countries specialised home care is implemented for a larger number of patients with heart failure [3]. In some countries mainly patients in the end-stage of life receive home care, while in other countries many patients with heart failure receive general home help with activities of daily living not specifically targeted at heart failure.

The guide was developed as part of a Seventh Framework European Union project (Homecare 222954, http://www.integratedhome.eu). The mission of the home care project was to investigate the benefits of integrated home care. Learning from successful projects in care for stroke patients, a special work package dedicated to explore the possibilities and the challenges for home care in heart failure and chronic obstructive pulmonary disease patients was initiated. This heart failure guide aims to summarise the knowledge and the experiences of home care as a service for heart failure patients to provide a description of the characteristics of home-based heart failure care and to develop guidance for establishing and delivering home-based heart failure care.

## Methods

In this guide, we defined home-based heart failure care as ‘care delivered with regard to heart failure management to a patient with sessions in the patient's own home’. Data from patient perspectives, health care providers and partners of patients are included. The guide is based on following sources:

- Literature review on the components in home care programmes [13].
- Survey of European heart failure management programmes [14].
- Opinion of researchers and practitioners in the field to evaluate the first draft of the components for optimal home-based management.
- Data from a clinical trial implementing the components for optimal home-based management in Sweden
- Data from retrospective analysis of 45 heart failure patients receiving home-based care in the Netherlands.
- Two-day expert meeting with international representatives (Sweden, Netherlands and Denmark)

## Results

### Which home-based heart failure care is available in Europe?

In a European survey [13], we described the organisation and components of care in 114 home-based management programmes for patients with heart failure in a selected number of European countries. If specialised heart failure home care was available, this was often provided by a multidisciplinary team, most often with nurses and physicians. Increased accessibility to care was provided by early follow-up (79%) as well as long-term follow-up (96%), telephone support (54%), telephone access (86%) and referral to remote monitoring (37%). In terms of more advanced treatment, 38% of the programmes could provide intravenous diuretics and most centres could refer their patients for device treatment. More than half of the programmes stated that they provided palliative care (67%) and end-of-life care (61%) in their programmes and 38% had joint care plans between primary and secondary care. Almost all programmes provided written education to the patients and to some extent to their families, the rest of the centres provided written education to the patients. And there seems a continuous interest to improve educational and self-care material [15,16].The components rated as most important by the health care professionals are shown in Table 1.

### Which patient may benefit from home-based heart failure care?

Although several randomised controlled studies are performed in heart failure management, they have not recruited all types of heart failure patients. On the one hand, most trials apply quite wide inclusion criteria regarding severity of heart failure, co-morbidity and have no upper age limit. On the other hand, the frailest patients more often refuse participation due to fatigue and poor health and health care providers may more or less purposively not ask the frailest patients for study participation. Although we realise that non-participation in trials does not automatically transfer to suitability of a home care intervention, recruitment problems to home care trials may give some indication on the suitability and feasibility of home care programmes or integrative programmes for heart failure patients.

Stewart and colleagues recently discussed the difficulty in recruitment in their study [11] which they assign to ‘entrenched preferences from a consumer and health professional perspective for each approach’, reflecting that some patients prefer being visited in the home (particularly if they are frail and having difficulty attending a hospital clinic). On the other hand, some patients actually prefer the idea of leaving their house, talking to other patients and seeing a range of health care workers at their local hospital.

**Figure 1.**
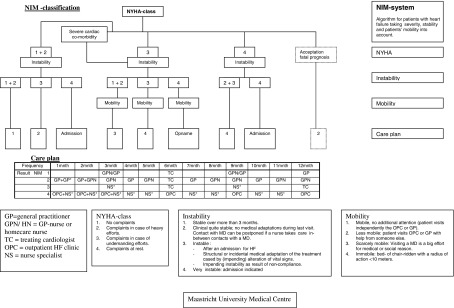
Example of classification (NIM) to use in determining the mode of care.

Not all patients may benefit from the same intervention. It is of great importance to find the optimal intervention for an individual patient and his/her family [16]. Eventually, it is not a matter of which professional at which place (home or hospital) who provides the best care for the heart failure patient, but rather how we can provide patients with integrated care in which health care professionals collaborate with each other in providing best care that fits patients in different situations and in different conditions [17]. It seems difficult to define exact criteria of which patients are suitable candidates for home care. Patients who are not able to travel to the heart failure clinic or to the hospital seem most appropriate candidates for heart failure care at home, mostly being frail patients. In a previous model in Maastricht (the Netherlands), the so-called NIM classification is used where NIM stands for a score calculated based on New York Heart Association-classification, Instability and Mobility (see Figure 1) [18]. In addition, patients' preferences need to be considered and an integrative model might be able to provide care that respects patient preferences for either home care, hospital care or both in a way that is feasible and cost-effective for the health care system.

### What are the considerations when implementing home-based heart failure care?

Home care for heart failure patients can both be seen as a supplement to hospital-based heart failure care or as a replacement. This means that in a lot of health care systems patients will able to use both home care and hospital-based care or that home care will be provided at certain times, while at other times hospital-based care is more in focus. Several organisational and structural issues, partner participation and staffing issues need to be considered.

#### Organisational and structural considerations

A common complaint, even in well-run services, is the lack of continuity between hospital and home or the lack of communication. Several programmes introduce a specific care plan or clinical pathways to improve care [13], but it is not always clear if the care plan is integrated between primary and secondary care. A specific description of the goals of the care and the responsibilities can help to improve quality of care [19,20]. Transition of care might improve inter-sector linkages using a joint care plan, for example, by including a nursing transfer letter, a telephone outreach, a notification of who is responsible for the care and a patient-held documentation [21].

At this moment, there are no evidence-based pathways published that describe the organisation of the care for heart failure patients. However, there are experiences with different ways of organising home-based care for heart failure patients. There are pathways based on structure or on patient's status. For example, a pathway based on structure can describe exactly when the patient will see a certain health care professional and locations might differ, for example, some care will be delivered in the home and other in the hospital. A pathways based on the patient's status might describe which care a patient receives depending on status, for example, a patients will have extra home visits in case of more or worsening symptoms.

#### Perspectives of patient and caregivers

Participation among heart failure patients who receive structured home care differs between patients. Home visits facilitate communication between the patient and caregiver and enables participation. Getting information is an important condition for participation that often is met during home visits. Good accessibility to care increases feeling of participation as well as the possibility to meet caregivers whom patients and caregivers trust. Several patients feel that a complex situation with several health care contacts limits the opportunity for participation [22].

#### Staffing issues: team members, qualifications, skills and knowledge

There is no uniform description of the specialisation or type of background of staff involved in home care for heart failure patients. For example, nurses can be home care nurses, hospital nurses, heart failure nurses, cardiac rehabilitation nurses, research nurses, practice nurses and/or district nurses [13]. Physicians can be cardiologists and/or primary care physicians or other specialists such as geriatricians or internists [13]. Two studies specifically reported collaboration between the primary care physician and cardiologist [23,24]. In one study, a team existed that consisted of a trained doctor's assistant and a primary care physician [25]. In another study, a team existed that consisted of a physician, a physiotherapist, an electrocardiography technician and a psychologist. In these teams, nurses were not involved [26]. Additionally, other professionals (psychologist, dietician, physical therapist, social worker and pharmacist) also often are involved in heart failure home care programmes [13], mostly as members of the multidisciplinary team or occasionally as the main provider of an intervention, for example, a pharmacist.

In general, specific training of the team members is not well described [13]. Specific training is mostly described in general terms such as, for example, or ‘trained nurses’ or ‘a one-day training course’ [13] and in some studies, some more information is provided such as the main aim of a programme or the description of theory and practice components [13]. The Heart Failure Association and the Council of Cardiovascular Nurses and Allied Professions of the European Society of Cardiology produced a training programme for a specialised nurse with expert knowledge of heart failure and its treatment, who can be part of a multidisciplinary team, and who has a holistic view of the patient throughout the continuum of care. Although this education is not specifically designed for integrative or home care professionals, it will provide a solid base to deliver optimal care. During the last 4 years this curriculum was implemented and tested in Sweden, Norway, Germany and Ireland [27].

### Implementing home-based heart failure care

During the projects in Sweden and the Netherlands, several barriers were found that might hinder implementation of home-based care. These were related to a need for ‘change of care culture’ that is to give more proactive care and treatment and the feeling that integrated care needs further development. Sometimes, unnecessary borders and barriers exist, and there is little clarity about responsibilities between different caregivers.

In addition, poor documentation and difficulty for nurses to assess and follow progress of symptoms and self-care make implantation of a new integrative model difficult. We also found that implementing a new model costs time and that the model may not fit all patients.

Not all health care professionals might be interested in implementing a new care model and a lack of skills among health care professionals is a challenge. Finally, reimbursement might not be possible for all care provided, and small teams can have difficulty to maintain continuity.

Barriers that exist to broadly implement integrated care in Europe might be different from country to country and be related to funding of health care services. At the same time, we face the challenge to make a shift in thinking from clinic-based care to care in the home. Currently, most heart failure disease management is still located in the hospitals [13], and health care professionals find it difficult to ‘let the patient go’, since they might feel this vulnerable population might not do well without highly specialised medical follow-up. This is a constant debate and recent evidence shows that specialists need to rethink the optimal location of care, including home care [28,29]. From a recent Swedish study, it was found that participants described that follow-up methods in integrative care were home visits, telephone support, video telephony, e-mail and Internet tools. Among concerns using a lot of information and communication technology tools were issues such as lack of personal contact with patient, the lack of information and communication technology knowledge among elderly, current information and communication technology systems not working properly [30].

Facilitators: In the Swedish and Dutch projects the following facilitators of implementation were described: First, ongoing education and skill building is important during the implementation phase to motivate staff and increase competence. Second, checklists and numeric rating scales for symptom evaluation were perceived as helpful. Positive aspects of a model can be stressed during implementation: better communication, better optimisation of treatment due to the model, possibility to change practice, actively involved next of kin and improved documentation

## Recommendations

From a previous cost analysis of integrated care for heart failure patients, we concluded that the value of outcomes supersedes the intervention costs, and integrated home care is expected to be health economic dominant, which gives some flexibility for local negotiation of adapted win-win solutions [12,30]. National health systems, statutory health insurances or private health insurances should consider the option of reimbursement of home care for heart failure patients and closely investigate pragmatic barriers of finance, for example, contradicting local financial incentives and interdisciplinary barriers within and across settings. The solution of barriers varies from region to region within the European Union.

From expert recommendation, the survey on existing components and pilot results it can be concluded that in integrating hospital and home care it is advised to consider the following components of home care for heart failure patients.

### Integrated, multidisciplinary care

- Integrate the care between community care, secondary and primary care
- Use a team approach
- Prioritise continuity of care and of staff members
- Decide the right level of care according to severity and stability of heart failure and other care needs
- Develop a communication plan within the team
- Include competent staff
- Staff should have specialised knowledge on heart failure care
- General courses or modules of courses that focus on heart failure according to the curriculum of Heart Failure training programme of the Heart Failure Association of the European Society of Cardiology are suitable for team members that deliver care to heart failure patients
- Guarantee assessment and documentation skills of staff

### Patient and partner participation

- Ask patients about their preferences of treatment and care
- Increase patients participation in care and increase partnership in care
- Use a Holistic approach to all patients and include co-morbid conditions
- Support close relatives

### Care plans with clear goals of care

- Make a care plan for every patient
- Individualise follow-up in a care plan with goals
- Make clear goals of care focussing on active and/or palliative treatment
- Focus care to improve quality of life, functional status and sense of security for patients
- Standardise assessment and documentation of assessment
- Include communication protocols

### Patient education

- Individualise patient education and built on previous knowledge
- Combine different types of media for patient education can be used
- Plan, repeat and document educational activities
- Include family in education

### Self-care management

- Apply Interventions to improve self-care
- Consider the use of alert system to detect early symptoms of deterioration
- Consider the use of tele-rehabilitation, tele-monitoring and telephone follow-up

### Appropriate access to care

- Adapt access to care to be patient and family needs
- Consider the use of tele-rehabilitation, tele-monitoring and telephone follow-up
- Use a palliative care approach were needed

### Optimise treatment

- Use guidelines to optimise treatment
- Define clear strategies for up-titrations of drugs and set clear indications for lab tests related to drug titration and deterioration
- Evaluate treatment and document the evaluation
- Individualise treatment

## Conclusion

Integrated home care should be seriously considered as an option towards the demographic ageing effect on health care in nearly all industrialised countries. In integrated home care for heart failure patients, it is advised to consider the following components: integrated, multidisciplinary care, patient and partner participation, care plans with clear goals of care, patient education, self-care management, appropriate access to care and optimised treatment.

## Reviewers

**Lynda Blue**, Regional Service Development Manager, British Heart Foundation, Scotland and Northern Ireland, London, UK.

**Helmut Hildebrandt**, CEO OptiMedis AG & Gesundes Kinzigtal GmbH, Germany.

**Mary Ryder**, Lecturer, School of Nursing & Midwifery, Edith Cowan University, Perth, Western Australia.

## Figures and Tables

**Table 1.**
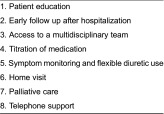
Components rated as most important (*n* = 114), presented in order with the top score component first
